# Thirty-Day Outcomes of Children and Adolescents With COVID-19: An International Experience

**DOI:** 10.1542/peds.2020-042929

**Published:** 2021-05-28

**Authors:** Talita Duarte-Salles, David Vizcaya, Andrea Pistillo, Paula Casajust, Anthony G. Sena, Lana Yin Hui Lai, Albert Prats-Uribe, Waheed-Ul-Rahman Ahmed, Thamir M. Alshammari, Heba Alghoul, Osaid Alser, Edward Burn, Seng Chan You, Carlos Areia, Clair Blacketer, Scott DuVall, Thomas Falconer, Sergio Fernandez-Bertolin, Stephen Fortin, Asieh Golozar, Mengchun Gong, Eng Hooi Tan, Vojtech Huser, Pablo Iveli, Daniel R. Morales, Fredrik Nyberg, Jose D. Posada, Martina Recalde, Elena Roel, Lisa M. Schilling, Nigam H. Shah, Karishma Shah, Marc A. Suchard, Lin Zhang, Ying Zhang, Andrew E. Williams, Christian G. Reich, George Hripcsak, Peter Rijnbeek, Patrick Ryan, Kristin Kostka, Daniel Prieto-Alhambra

**Affiliations:** a Fundació Institut Universitari per a la Recerca a l’Atenció Primària de Salut Jordi Gol i Gurina, Barcelona, Spain; b Bayer Pharmaceuticals, Sant Joan Despi, Spain; c Real-World Evidence, Trial Form Support, Barcelona, Spain; d Janssen Research & Development, Titusville, New Jersey; e Department of Medical Informatics, Erasmus University Medical Center, Rotterdam, Netherlands; f School of Medical Sciences, University of Manchester, Manchester, United Kingdom; g Centre for Statistics in Medicine, Nuffield Department of Orthopaedics, Rheumatology and Musculoskeletal Sciences; m Nuffield Department of Clinical Neurosciences, University of Oxford, Oxford, United Kingdom; h College of Medicine and Health, St Luke’s Campus, University of Exeter, Exeter, United Kingdom; i Medication Safety Research, King Saud University, Riyadh, Saudi Arabia; j Faculty of Medicine, Islamic University of Gaza, Gaza, Palestine; k Massachusetts General Hospital and Harvard Medical School, Harvard University, Boston, Massachusetts; l Department of Biomedical Informatics, School of Medicine, Ajou University, Suwon, South Korea; n Department of Veterans Affairs, Salt Lake City, Utah; o School of Medicine, University of Utah, Salt Lake City, Utah; p Department of Biomedical Informatics, Columbia University, New York, New York; q Regeneron Pharmaceuticals, Tarrytown, New York; r Department of Epidemiology, Johns Hopkins Bloomberg School of Public Health, Johns Hopkins University, Baltimore, Maryland; s DHC Technologies, Co, Ltd, Beijing, China; t Lister Hill National Center for Biomedical Communications, National Library of Medicine, National Institutes of Health, Bethesda, Maryland; u Bayer AG, Wuppertal, Germany; v Division of Population Health and Genomics, School of Medicine, University of Dundee, Dundee, United Kingdom; w School of Public Health and Community Medicine, Institute of Medicine, Sahlgrenska Academy, University of Gothenburg, Gothenburg, Sweden; x Department of Medicine, School of Medicine, Stanford University, Stanford, California; y Universitat Autònoma de Barcelona, Barcelona, Spain; z Data Science to Patient Value Program, Department of Medicine, School of Medicine, University of Colorado Anschutz Medical Campus, Aurora, Colorado; aa Department of Biostatistics, Fielding School of Public Health, University of California, Los Angeles, Los Angeles, California; bb School of Population Medicine and Public Health, Peking Union Medical College and Chinese Academy of Medical Sciences, Beijing, China; cc Melbourne School of Population and Global Health, The University of Melbourne, Melbourne, Australia; dd Institute for Clinical Research and Health Policy Studies, Tufts Medical Center, Boston, Massachusetts; ee Real World Solutions, IQVIA, Cambridge, Massachusetts

## Abstract

**OBJECTIVES:**

To characterize the demographics, comorbidities, symptoms, in-hospital treatments, and health outcomes among children and adolescents diagnosed or hospitalized with coronavirus disease 2019 (COVID-19) and to compare them in secondary analyses with patients diagnosed with previous seasonal influenza in 2017–2018.

**METHODS:**

International network cohort using real-world data from European primary care records (France, Germany, and Spain), South Korean claims and US claims, and hospital databases. We included children and adolescents diagnosed and/or hospitalized with COVID-19 at age <18 between January and June 2020. We described baseline demographics, comorbidities, symptoms, 30-day in-hospital treatments, and outcomes including hospitalization, pneumonia, acute respiratory distress syndrome, multisystem inflammatory syndrome in children, and death.

**RESULTS:**

A total of 242 158 children and adolescents diagnosed and 9769 hospitalized with COVID-19 and 2 084 180 diagnosed with influenza were studied. Comorbidities including neurodevelopmental disorders, heart disease, and cancer were more common among those hospitalized with versus diagnosed with COVID-19. Dyspnea, bronchiolitis, anosmia, and gastrointestinal symptoms were more common in COVID-19 than influenza. In-hospital prevalent treatments for COVID-19 included repurposed medications (<10%) and adjunctive therapies: systemic corticosteroids (6.8%–7.6%), famotidine (9.0%–28.1%), and antithrombotics such as aspirin (2.0%–21.4%), heparin (2.2%–18.1%), and enoxaparin (2.8%–14.8%). Hospitalization was observed in 0.3% to 1.3% of the cohort diagnosed with COVID-19, with undetectable (n < 5 per database) 30-day fatality. Thirty-day outcomes including pneumonia and hypoxemia were more frequent in COVID-19 than influenza.

**CONCLUSIONS:**

Despite negligible fatality, complications including hospitalization, hypoxemia, and pneumonia were more frequent in children and adolescents with COVID-19 than with influenza. Dyspnea, anosmia, and gastrointestinal symptoms could help differentiate diagnoses. A wide range of medications was used for the inpatient management of pediatric COVID-19.

Since January 2020, a growing number of infections by the severe acute respiratory syndrome coronavirus 2 (SARS-CoV-2) has led to an unprecedented pressure on health care systems worldwide. Coronavirus disease 2019 (COVID-19) affects all age groups, with pediatric population representing 3.7% of reported cases.^1^

Despite children and adolescents being more susceptible to certain infectious diseases because of their developing immune system,^2^ clinical manifestations of COVID-19 are generally milder in the pediatric population,^3,4^ with better outcomes and lower mortality rates than adults.^5^ Nevertheless, there is evidence of children and adolescents with COVID-19 requiring hospitalization and ICU-level care. Reports from the United States or China revealed that a low number of pediatric COVID-19 cases were hospitalized (5.7%)^6^ or admitted to the ICU (1.8%),^7^ respectively. However, in a study conducted in 25 European countries, researchers found that in a sample of 582 children and adolescents, 63% were hospitalized.^8^ Because the data included in this last study were limited to April 2020, the high admission rate reported could reflect temporal trends in testing availability because only the most ill children might have been tested during this period.

To date, most clinical guidelines recommend supportive care as the mainstay of therapy in children,^9–11^ but there are few data to recommend or reject the use of specific immunomodulatory drugs or antiviral agents, particularly from clinical trials in the pediatric population.^12^ It also remains to be elucidated whether children and adolescents show a different clinical presentation.^13^ We conducted a literature review for articles published in PubMed and medRxiv between December 2019 and June 2020 in which authors reported on patients with a confirmed COVID-19 diagnosis. Of the 1320 studies that met the inclusion criteria, only 79 studies were on children and adolescents, most of which (63%) were local case reports or case series. This compellingly reveals a large remaining gap in existing efforts to define the characteristics of the pediatric population in a real-world setting at a large scale.

In this study, we aimed to describe the demographics, comorbidities, symptoms, in-hospital treatments, and health outcomes of children and adolescents diagnosed or hospitalized with COVID-19 in the United States, Europe, and Asia. In addition, we compared these cohorts with children and adolescents diagnosed with seasonal influenza in 2017–2018 as a benchmark in a secondary analysis.

## METHODS

### Study Design, Setting, and Data Sources

This study is part of the Characterizing Health Associated Risks, and Your Baseline Disease In Severe acute respiratory syndrome coronavirus 2 (CHARYBDIS) study, a large-scale multinational cohort study using routinely collected primary care and hospital electronic health records (EHRs), hospital billing data, and insurance claims data from the United States, Europe (Netherlands, Spain, the United Kingdom, Germany, and France), and Asia (South Korea and China).

From the 19 databases contributing data to CHARYBDIS, only those with data on patients <18 years of age with a clinical diagnosis of COVID-19 or test result positive for SARS-CoV-2 between January and June 2020 were included. A cohort of children and adolescents diagnosed with seasonal influenza in 2017–2018 was included for comparison.

**FIGURE 1:**
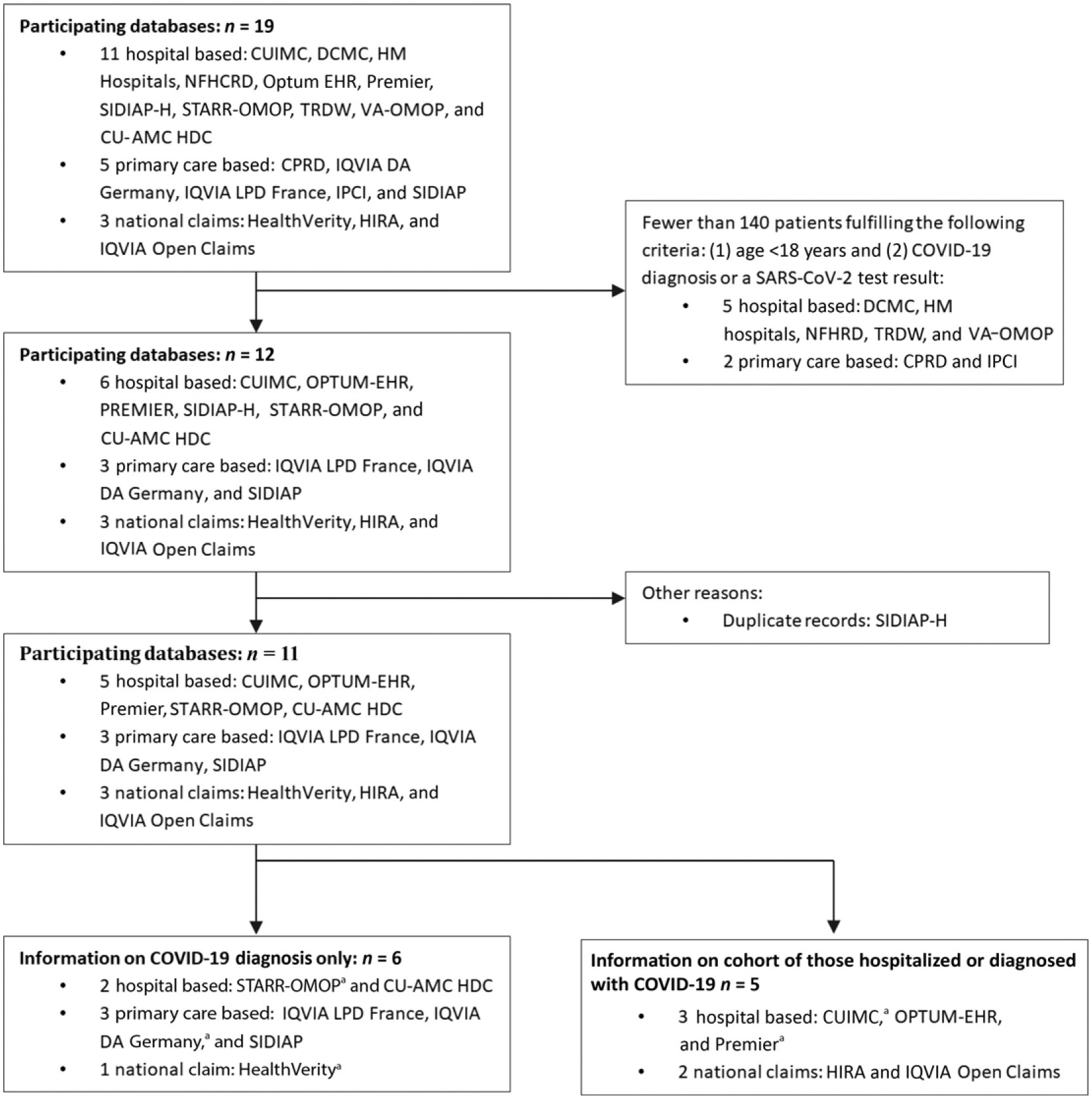
Database selection process for CU-AMC HDC, CUIMC, Clinical Practice Research Datalink (CPRD), DA, Daegu Catholic University Medical Center (DCMC), HIRA, Integrated Primary Care Information (IPCI), LPD, Nanfang Hospital COVID-19 Research Database (NFHCRD), the Information System for Research in Primary Care (SIDIAP), STARR-OMOP, Tufts Research Data Warehouse (TRDW), and the Department of Veterans Affairs Observational Medical Outcomes Partnership (VA-OMOP). HM, Hospitales de Madrid; SIDIAP-H, System for Research in Primary Care Hospitales. ^a^ No previous observation time was available; therefore, the data were excluded from the description of comorbidities.

To be included in the study, databases had to have a minimum of 140 children and adolescents. This cutoff was deemed necessary to estimate with sufficient precision (confidence interval width of ±5%) the prevalence of a previous condition or 30-day risk of an outcome affecting 10% of the study population. Data results for this article were extracted from CHARYBDIS results on October 1, 2020. The selection process of the databases for this study is shown in Fig 1. Eleven databases fulfilled the inclusion criteria: STAnford Research Repository Observational Medical Outcomes Partnership (STARR-OMOP) (United States),^14^ Colorado University Anschutz Medical Campus Health Data Compass (CU-AMC HDC) (United States), HealthVerity (United States), Columbia University Irving Medical Center (CUIMC) (United States), Optum EHR (United States), Premier Healthcare Database (United States), IQVIA Open Claims (United States), IQVIA Longitudinal Patient Data (LPD) France (France), IQVIA Data Analyzer (DA) Germany (Germany), Information System for Research in Primary Care (SIDIAP) (Spain),^15^ and Health Insurance Review & Assessment Service (HIRA) (South Korea). Among these, 5 databases contributed to the hospitalized cohort: Premier, Optum EHR, IQVIA Open Claims, CUIMC, and HIRA; and 3 were national claims databases: HealthVerity, HIRA, and IQVIA Open Claims. A more detailed description of the included data sources is available in Supplemental Table 2.

### Study Participants and Follow-up

Two nonmutually exclusive cohorts were included: (1) children and adolescents with a COVID-19 diagnosis or a SARS-CoV-2–positive test result (index date was the first of the 2 events) and (2) children and adolescents hospitalized (index date was hospitalization date) with a COVID-19 diagnosis or a SARS-CoV-2–positive test result 21 days before or after hospitalization date. A similar diagnosed cohort of children and adolescents with seasonal influenza diagnosis or positive influenza test result in 2017–2018 was also included. Study participants could contribute information to all cohorts (diagnosed or hospitalized with COVID-19 or diagnosed with influenza). Individuals diagnosed with COVID-19 could be part of the hospitalized cohort if COVID-19 was diagnosed during or at the time or 21 days before hospitalization. Cohort participants were managed in each cohort from the index date to the earliest of death, end of the observation period, or 30 days after. The codes used to identify COVID-19 are described in Supplemental Table 3. To describe history of comorbidities, only participants with at least 365 days of previous observation before the index date were included. Children <1 year of age were excluded from the cohort databases for which we required 365 days of previous observation.

### Baseline Characteristics, Symptoms, Drug Use, and Outcomes of Interest

Baseline information on age at the index date and conditions up to 1 year before the index date were identified. Conditions were ascertained on the basis of the Systematized Nomenclature of Medicine–Clinical Terms hierarchy, with all descendant codes included. Detailed definitions of each condition can be consulted in Supplemental Table 3.

Symptoms recorded at the index date included fever; cough; dyspnea; malaise or fatigue; myalgia; anosmia, hyposmia, or dysgeusia; gastrointestinal tract symptoms; diarrhea; vomiting; and nausea. All drugs prescribed or dispensed during the 30-day follow-up after the index date were ascertained. Individual medications were categorized by using Anatomical Therapeutic Chemical groupings. For the study of medications potentially used for COVID-19, we assessed all medications included in at least 2 randomized controlled trials.^16^ The list was further enriched with suggestions from key stakeholders including regulatory agencies, key opinion leaders, and the pharmaceutical industry. Medicines of interest were grouped into (1) repurposed medications (those with alternative indications but believed to be efficacious as antiviral agents) and (2) adjuvant therapies (used allegedly for the treatment or prevention of COVID-19 complications).^17^ All conditions and medications and additional time windows (a month before and on the index date) are reported in full and are available in an interactive Web site: https://data.ohdsi.org/Covid19CharacterizationCharybdis/.

The 30-day outcomes described in the diagnosed cohorts included hospitalization, death, pneumonia, and multisystem inflammatory syndrome in children (MIS-C) (including Kawasaki disease or toxic shock syndrome). In the hospitalized cohorts, we additionally report sepsis, total cardiovascular disease events, acute respiratory disease syndrome (ARDS), cardiac arrhythmia, and bleeding. The definition of each outcome is provided in Supplemental Table 3.

We used the Observational Health Data Sciences and Informatics Cohort Diagnostics to assess the fitness of use of each comorbidity per database. This tool represents the codes used for the definition of each condition and the prevalence of these conditions by sex, age groups, and calendar year for each database. Results from cohort diagnostics of the definitions of COVID-19, seasonal influenza, and comorbidities are publicly available at https://data.ohdsi.org/Covid19CharacterizationCharybdisDiagCovid/, https://data.ohdsi.org/Covid19CharacterizationCharybdisDiagInfluenza/, https://data.ohdsi.org/Covid19CharacterizationCharybdisDiagFeature/, and https://data.ohdsi.org/Covid19CharacterizationCharybdisDiagStrata/.

### Statistical Analyses

All data were standardized to the Observational Medical Outcomes Partnership Common Data Model.^18^ A common analytical code for the CHARYBDIS study was developed for the Observational Health Data Sciences and Informatics methods library, which was run locally in each database. Only aggregate results from each database were publicly shared. The CHARYBDIS protocol and source code can be found at https://github.com/ohdsi-studies/Covid19CharacterizationCharybdis. Results were extracted from CHARYBDIS on October 1, 2020.

Demographics, history of comorbidities, symptoms, and outcomes were summarized as proportions, calculated by dividing the number of people within a given category by the total number of people in each specific cohort. The proportion of users of each medication was determined for the hospitalized children and adolescents as the percentage of subjects who had ≥1 day during the time window overlapping with a drug use period for each medication or drug class of interest. The use of repurposed and adjuvant drugs up to 30 days after admission was described.

The distribution of conditions a year before index, symptoms at index, outcomes, and medications up to 30 days post–index date in the COVID-19–diagnosed cohort were compared with the hospitalized COVID-19 cohort or the influenza-diagnosed cohort. Standardized mean differences were calculated when comparing the characteristics of study cohorts.

We performed a sensitivity analysis describing characteristics of cases with no previous observation time to understand the impact of the lack of previous observation time in the results.

We used R version 3.6 (R Foundation for Statistical Computing, Vienna, Austria) for data visualization. All the data partners obtained institutional review board approval or exemption to conduct this study, as required.

All the data partners received institutional review board approval or exemption.

## RESULTS

A total of 242 158 children and adolescents diagnosed (9769 hospitalized) with COVID-19 and 2 084 180 diagnosed with seasonal influenza were included. Data were obtained from 5 US hospital EHR databases (STARR [with the pediatric population representing 3.9% of all COVID-19 cases in this database], CU-AMC HDC [2.6% of all COVID-19 cases], CUIMC [4.1% of all COVID-19 cases], Optum EHR [5.7% of all COVID-19 cases], and Premier [5.1% of all COVID-19 cases]); 3 European primary care records databases (IQVIA LPD France [4.0% of all COVID-19 cases], IQVIA DA Germany [10.0% of all COVID-19 cases], and SIDIAP-Spain [3.7% of all COVID-19 cases]); and 2 US (HealthVerity [4.6% of all COVID-19 cases], IQVIA Open Claims [6.8% of all COVID-19 cases]) and 1 Asian claims databases (HIRA–South Korea [3.3% of all COVID-19 cases]). Up to at least 1 year of preindex observation time was available only in CU-AMC HDC, Optum EHR, SIDIAP-Spain, and IQVIA Open Claims. Figure 1 reveals a flowchart outlining the reasons for exclusion of additional 8 data sources available in CHARYBDIS.

### Demographics

The age at diagnosis of COVID-19 varied across regions. In SIDIAP-Spain, CUIMC, Optum EHR, Premier, and STARR, the majority of children with COVID-19 were diagnosed with the COVID-19 at ages 0 to 4 years (approximately one-third), whereas the proportion of children from 0 to 4 years was only 11.4% and 11.6% in IQVIA LPD France and CU-AMC HDC, respectively. More consistently, most of the hospital admissions were seen in the younger groups (0–4 years) (eg, 57.1% of those hospitalized in CUIMC and 54.2% in Premier). Male sex was more common in all databases except for in IQVIA LPD France (46.6% male) and STARR (49.2%) (Table 1).

### Comorbidities

**FIGURE 2:**
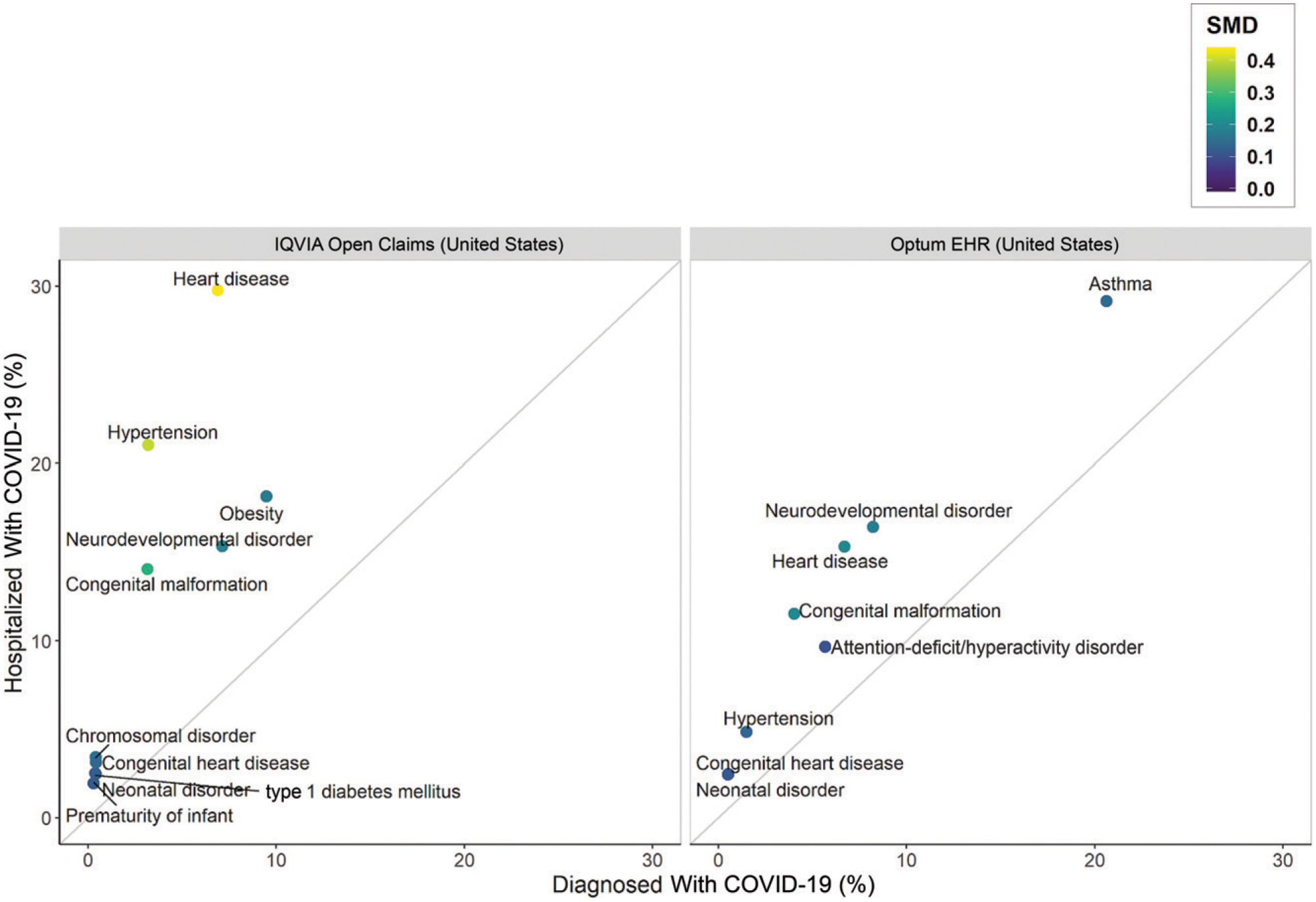
Prevalence of previous comorbidities among children and adolescents (<18 years of age) diagnosed with COVID-19 (x-axis) compared with those hospitalized (y-axis) with COVID-19. Hospitalized children and adolescents could also be included in the diagnosed cohorts. SMD, standardized mean difference in prevalence between the x- and y-axes.

In patients diagnosed with COVID-19, asthma was the most common baseline comorbidity assessed in the year before the index date, affecting 10.1% (SIDIAP-Spain) to 28.1% (IQVIA Open Claims), followed by obesity (from 1.9% in IQVIA LPD France to 19.0% in Optum EHR). Also in the cohort of individuals diagnosed with COVID-19, we observed a high prevalence of congenital malformation(s) (3.2% of those diagnosed with COVID-19 in IQVIA Open Claims to 10.8% in SIDIAP-Spain), neurodevelopmental disorders (1.0% of those diagnosed with COVID-10 in IQVIA LPD France to 8.2% in Optum EHR), heart disease (1.2% in IQVIA LPD France to 6.9% in IQVIA Open Claims), type 1 diabetes mellitus (0.2% in SIDIAP-Spain to 0.4% in IQVIA Open Claims), cancer (0.3% in SIDIAP-Spain to 3.4% in Optum EHR), and chromosomal disorder(s) (0.4% in SIDIAP-Spain to 0.5% in Optum EHR). All of these were more common among hospitalized children and adolescents with COVID-19 as compared with the cohort diagnosed with COVID-19 (standardized mean difference >0.1; assessed in the year before the index date): 34.1% asthma, 18.0% obesity, 29.7% heart disease (3.0% congenital heart disease), 9.3% cancer, 3.4% chromosomal disorder(s), 14.0% congenital malformation(s), and 2.0% prematurity in IQVIA Open Claims (Fig 2).

### Symptoms

**FIGURE 3:**
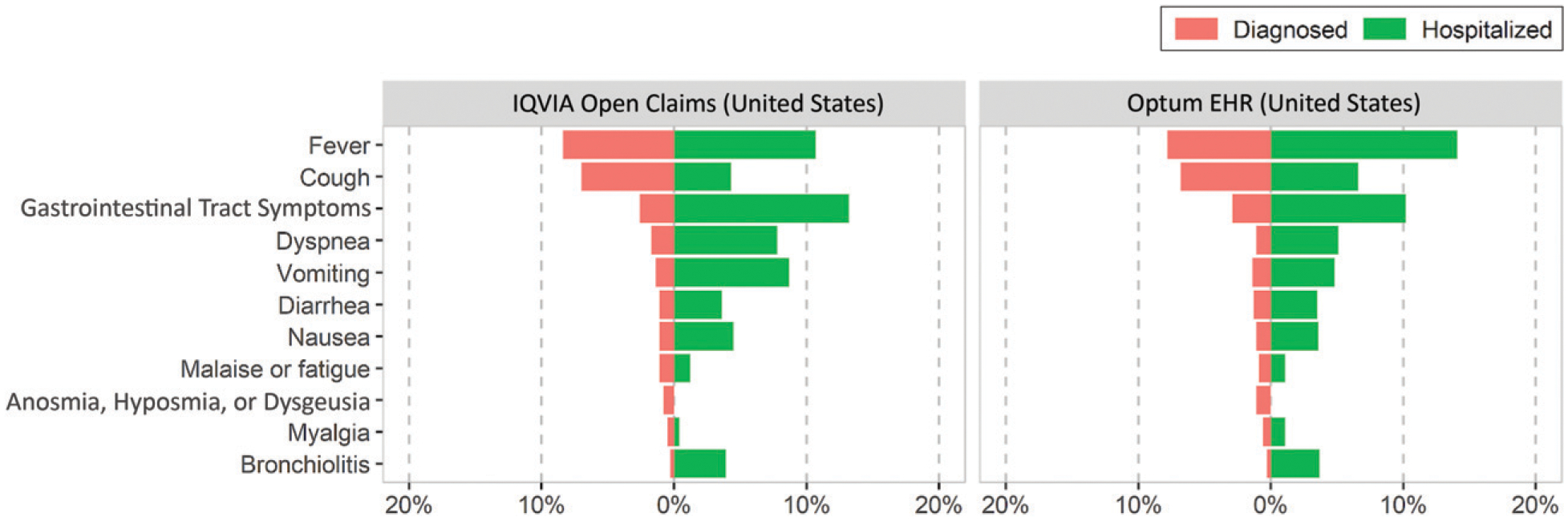
Symptoms recorded at the index date among children and adolescents (<18 years of age) diagnosed with COVID-19 compared with those hospitalized with COVID-19. Hospitalized children and adolescents could also be included in the diagnosed cohorts.

Figure 3 reveals the recorded symptoms at the index date for diagnosed versus hospitalized patients with COVID-19. The most common reported symptom in COVID-19 was fever, seen in 4.8% (SIDIAP-Spain) to 26.4% (CUIMC) of patients diagnosed with COVID-19, and higher (up to 28.1% in CUIMC) among hospitalized patients. The second most common was cough, recorded in 4.7% (SIDIAP-Spain) to 13.0% (Premier) among patients diagnosed with COVID-19, and lower (eg, 2.2% in Premier) in hospitalized children and adolescents. Bronchiolitis was recorded in 0.5% (SIDIAP-Spain) to 9.7% (STARR) of patients diagnosed with COVID-19 and higher (up to 14.4% in Premier) in the hospitalized. Gastrointestinal tract symptoms were also common at the index date, recorded in 0.5% (HealthVerity) to 12.5% (SIDIAP-Spain) in patients diagnosed with COVID-19, and up to 13.2% (IQVIA Open Claims and Premier) among the hospitalized. Anosmia was ≤1% in all participating databases, except for patients diagnosed with COVID-19 in IQVIA Open Claims (1.1%) and Premier (1.5%). Compared with influenza (Supplemental Fig 6), children and adolescents diagnosed with COVID-19 had fewer frequently recorded symptoms by health care professionals in most databases, with the only exceptions of dyspnea (eg, 8.6% in COVID-19 versus 1.1% in influenza in STARR); bronchiolitis (eg, 9.7% in COVID-19 versus 1.1% in influenza in STARR); anosmia, hyposmia, or dysgeusia (eg, 0.8% in COVID-19 versus 0.0% in influenza in IQVIA Open Claims); and gastrointestinal tract symptoms (eg, 10.3% in COVID-19 versus 4.5% in influenza in IQVIA Open Claims).

### In-Hospital Treatments

**FIGURE 4:**
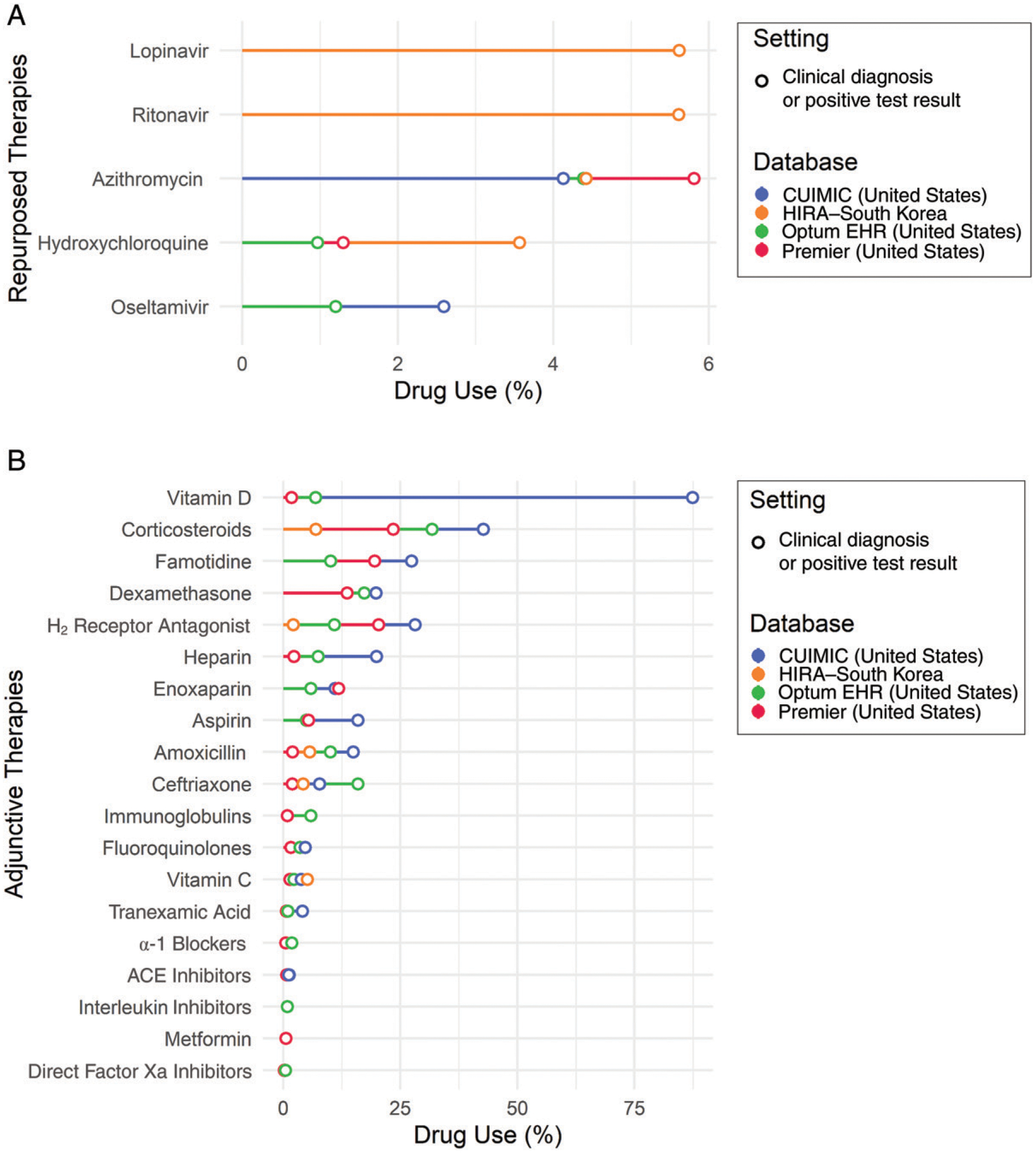
Thirty-day in-hospital use of treatments among children and adolescents (<18 years of age) with COVID-19. A, Repurposed drugs. B, Adjunctive therapies. ACE, Angiotensin converting enzyme; H_2_, histamine; Xa, xabans.

The use of drugs during hospital admission for COVID-19 among children and adolescents is reported in Fig 4A (repurposed) and Fig 4B (adjunctive therapies) on the basis of data from CUIMC, Optum EHR, Premier, and HIRA–South Korea. Repurposed treatments were not commonly used (<10% in all databases), with lopinavir-ritonavir used in 5.6% in HIRA–South Korea but not in US databases, azithromycin from 4.4% in HIRA–South Korea and Optum EHR to 5.8% to 5.8% in Premier, hydroxychloroquine in 1.0% (Premier) to 3.6% (HIRA–South Korea), and oseltamivir only in the United States, from 1.2% in Optum EHR to 5.6% in CUIMC.

Adjunctive therapies were more common, with systemic corticosteroids used in 6.8% (HIRA–South Korea) to 48.6% (CUIMC). Famotidine was the second most common adjunctive treatment, used in 10.0% (Optum EHR) to 28.1% (CUIMC). Concomitant antithrombotic therapy was also common in the United States but not in HIRA–South Korea (no use reported), including aspirin (4.8% in Premier to 21.4% in CUIMC), heparin (2.2% in Premier to 18.1% in CUIMC), and enoxaparin (6.0% in Optum EHR to 11.7% in Premier). Antibiotics (ceftriaxone, amoxicillin, and fluoroquinolones), vitamin (D and C) supplements, and immunoglobulins were also used with high variability between the contributing databases.

### Health Outcomes

**FIGURE 5:**
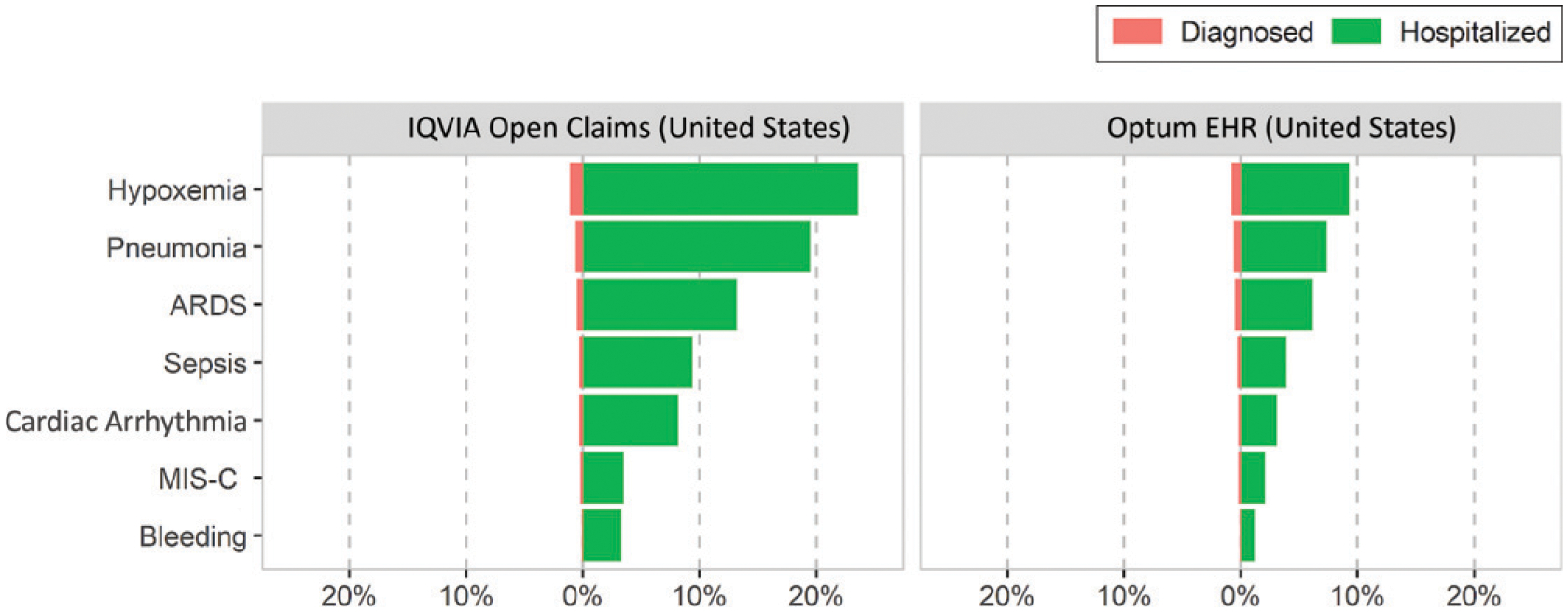
Main 30-day outcomes among children and adolescents (<18 years of age) diagnosed compared with hospitalized with COVID-19. Hospitalized children and adolescents could also be included in the diagnosed cohorts.

Outcomes in the 30-day period after the diagnosis of COVID-19 and hospitalization with COVID-19 are summarized in Table 1 and Fig 5. Hospitalization was observed in a low proportion (0.3% in HealthVerity and 1.4% in SIDIAP-Spain) in the ambulatory and claims databases with testing and testing results data available (in 3.5% in IQVIA Open Claims) and more frequent (7.6% in Optum EHR to 33.2% in CUIMC) in hospital EHR databases. Hypoxemia and pneumonia were the most common complications. Hypoxemia was diagnosed in a wide range from 0.1% (HealthVerity) to 13.5% (STARR) of those diagnosed with COVID-19 and in 12.9% (CUIMC) to 23.6% (IQVIA Open Claims) of those hospitalized with COVID-19. Pneumonia was diagnosed in a range from 0.1% (HealthVerity) to 4.5% (CUIMC) of those diagnosed with COVID-19 and in 6.8% (HIRA–South Korea) to 15.6% (IQVIA Open Claims) of those hospitalized with COVID-19. ARDS was the most common in-hospital outcome, affecting 6.2% (Optum EHR) to 16.5% (Premier) of those hospitalized with COVID-19. Sepsis during admission was observed in <2% (*n* < 5; HIRA–South Korea) to 10.1% in Premier. Other less common outcomes are reported in Table 1. MIS-C was seen in <0.1% (*n* < 5; SIDIAP-Spain) to 3.1% (CUIMC) among those diagnosed with COVID-19 and up to 7.6% (CUIMC) in hospitalized patients with COVID-19.

A comparison of outcomes in those diagnosed with COVID-19 versus those diagnosed with influenza in previous years is depicted in Supplemental Fig 7. Hospitalization rates were higher for COVID-19–diagnosed versus influenza-diagnosed children and adolescents (eg, 3.5% vs 0.9% in IQVIA Open Claims, 33.2% vs 7.4% in CUIMC, and 30.8% vs 3.7% in STARR), except in Optum EHR in which hospitalization rates were 7.6% in COVID-19–diagnosed and 10.6% in influenza-diagnosed participants. Similarly, hypoxemia was more common in COVID-19–diagnosed participants (eg, 13.5% vs 1.7% in STARR-OMOP and 23.6% vs 0.3% in IQVIA Open Claims), as well as pneumonia (eg, 3.1% vs 0.1% in SIDIAP-Spain and 1.8% vs 0.8% in IQVIA Open Claims), and, despite rare, MIS-C (eg, 0.2% vs 0.0% in IQVIA Open Claims and 3.1% vs <0.2% in CUIMC). Other outcomes had more similar risks in both viral infections (see Supplemental Fig 7 and Supplemental Table 4).

In a sensitivity analysis, we replicated the analyses including participants who had no previous history available in their EHRs (Supplemental Table 5). Differences in symptoms or outcomes were modest; however, they revealed the expected incompleteness in prevalent comorbidity.

## DISCUSSION

With this study, we comprehensively report on the largest cohort of children and adolescents with COVID-19 to date. Overall, most cases of COVID-19 diagnosis and related hospitalizations were seen among infants and toddlers aged <4 years old, predominantly of male sex. Children and adolescents hospitalized with COVID-19 had a higher prevalence of comorbidities than the overall cohort of those diagnosed with COVID-19, including asthma, obesity, heart disease, cancer, chromosomal disorder(s), and congenital malformation(s). The most commonly observed symptoms were fever and cough, whereas dyspnea, bronchiolitis, anosmia, and gastrointestinal tract symptoms were more common in children and adolescents with COVID-19 than with seasonal influenza and may aid on the differentiation of COVID-19 from other viral infections. The drug use analysis suggests little use of repurposed drugs and a substantial use of adjunctive therapies among children and adolescents.

Hospitalization rates were between fivefold and 13-fold higher among those children and adolescents diagnosed with COVID-19 versus those with seasonal influenza in previous years. Fortunately, 30-day fatality after a COVID-19 diagnosis or hospitalization was low. Respiratory complications were overrepresented among children and adolescents diagnosed with COVID-19 compared with seasonal influenza.

The proportion of children and adolescents <18 years of age from all observed COVID-19 cases in each database varied from 2.6% to 10.0%. These values are higher than what has been previously reported in other studies from China (2%),^19^ Spain (2%),^20^ the United States (1.7%),^6^ or the United Kingdom (0.9%)^21^ but are in line with the World Health Organization dashboard, which reported that children (>14 years) accounted for 3.7% of all COVID-19 cases,^1^ or the Australian Health Protection Principal Committee, which has reported that children and adolescents (<19 years) accounted for 4% of confirmed COVID-19 cases in Australia.^22^

Asthma and obesity were the most common baseline comorbidities in children and adolescents with COVID-19; this is in keeping with disease prevalence among a general pediatric population.^23^ More strikingly, we observed a high prevalence of conditions that are relatively rare in children and adolescents, including congenital malformation(s), neurodevelopmental disorders, heart disease, type 1 diabetes mellitus, cancer, and chromosomal disorder(s). These conditions were more frequent among hospitalized children and adolescents with COVID-19 than those diagnosed with COVID-19. This is in line with previous studies suggesting that children with comorbidity history have a higher risk of critical care admission.^21,24^

The frequency of reported symptoms in our study is generally lower than what has been previously reported in the pediatric literature,^8,21,24^ suggesting an underestimation in the register of symptoms in the form of structured data in busy actual care settings. An important finding is that children and adolescents diagnosed with COVID-19 presented with higher rates of dyspnea, anosmia, and gastrointestinal symptoms than children with seasonal influenza. This information is clinically relevant for differential diagnosis between COVID-19 and influenza among children and adolescents.

We observed great heterogeneity across countries in the use of in-hospital treatments among children and adolescents with COVID-19, which is in line with previous studies in adults.^25^ Our analysis suggests little use of antiviral therapies overall, with ~5% of children and adolescents hospitalized with COVID-19 using lopinavir-ritonavir in South Korea (but none in the United States), a variable proportion of use of oseltamivir (1%–5%) in the United States (but none in South Korea), a 4% to 6% use of azithromycin, and limited use of hydroxychloroquine, ranging from 1% to ~4% in both countries. These values are lower than what we previously reported in adults (eg, use of hydroxychloroquine was 57.9% in the overall population versus 1% in children and adolescents in Premier)^25^ but are in line with recent European cohort studies in hospitalized children and adolescents with COVID-19. In a study in the United Kingdom, researchers reported that 6% (38 of 591) of hospitalized children and adolescents with COVID-19 received antiviral drugs (30 received acyclovir, 7 received remdesivir, and 3 received chloroquine or hydroxychloroquine),^21^ whereas researchers in a study of 25 European countries including 582 children and adolescents found that 7% were treated with hydroxychloroquine, 3% with remdesivir, 1% with lopinavir-ritonavir, and 1% with oseltamivir.^8^ In contrast, we observed substantial use of different adjunctive therapies; corticosteroids were used in 25% to 35% in the United States but 7% in South Korea. Famotidine (2%–20%), aspirin (10%–30%), and vitamin D (2%–15%) were used in the United States but not in South Korea. Antibiotics were also commonly used, with ceftriaxone and amoxicillin among the most commonly prescribed. This is consistent with the previous study in the United Kingdom, where antibiotics were given to 69% (415 of 601) of hospitalized children with COVID-19.^21^

It is reassuring that occurrence of severe outcomes during the 30 days after diagnosis of COVID-19 was rare in our study, which is in line with previous studies.^8,20,21,24^ MIS-C was relatively uncommon, affecting 0.5% to 3.1% of all patients diagnosed with COVID-19 but up to 0.9% to 7.6% of those hospitalized with COVID-19. These results are in line with previous studies from Europe and the United States, which have suggested that COVID-19 may be associated with MIS-C in children.^26–28^ In a separate cohort study, researchers found recently that 11% of children with COVID-19 admitted to hospitals in the United Kingdom developed MIS-C.^21^ These findings are of special relevance given the severity of this condition. Overall, all outcomes were more frequent in children and adolescents with COVID-19 diagnosis than those with a diagnosis of seasonal influenza in 2017–2018, suggesting more severe disease prognosis in children with COVID-19 than influenza. Future research is needed to characterize and determine the long-term outcomes of children and adolescents affected with COVID-19.

This study has some limitations. First, this study is descriptive in nature. The observed differences between groups should therefore not be interpreted as causal effects. Second, our results are based on secondary data from electronic records collected for administrative and clinical management purposes and reused for research, which may affect the completeness of data recorded (eg, lack of information on the degree of prematurity or mortality) and may have erroneous entries, leading to potential misclassification. Such incompleteness could be differential in some instances (eg, hospital versus primary care settings) and risk information bias for the proposed comparisons. The underreporting of symptoms observed in these data is a key finding of this study and should be taken into consideration in previous and future similar reports from real-world cohorts. This could be due to the high workload in health care systems caused by the pandemic, coding practices, or difficulty of ascertainment of symptoms among preverbal children. Another limitation of this study is the inability to differentiate between children and adolescents tested for SARS-CoV-2 because of the presentation of symptoms versus those tested as part of surveillance campaigns. Finally, the currently available data are restricted to the first 6 months of 2020 coinciding with the peak of the COVID-19 pandemic in the studied countries; therefore, we were not able to evaluate the possible changes in treatment and prognosis over time.

This study is unique because our approach to characterizing children with an international scope allows for a wide range of variation in health care systems and policies against the COVID-19 pandemic. This enables a more complete understanding of the implications of the pandemic for different countries and regions in scope of an international comparison. It also poses a layer of heterogeneity that needs to be considered in the interpretation of our findings, opening a window for new research questions that need to be addressed, particularly around the public health approach for controlling the pandemic spread and severity in children and adolescents and overall. This study represents, to our knowledge, the largest cohort study on pediatric COVID-19 to date and the only study used to provide a comparison with children and adolescents with seasonal influenza in 2017–2018. Our data confirm low rates of complications in children with COVID-19, with severe cases clustering among children with previous comorbidities. Despite the large sample size available, MIS-C appears rare.

## CONCLUSIONS

COVID-19 affects children and adolescents of all ages, but severe outcomes are reassuringly uncommon. There is variability across health care systems in different regions of America, Asia, and Europe that may explain the observed differences in the epidemiology and clinical management of the disease as well as observed outcomes. Despite negligible fatality, complications including hospitalization, hypoxemia, and pneumonia are more frequent in children and adolescents with COVID-19 than with influenza. Dyspnea, anosmia, and gastrointestinal symptoms could help differential diagnosis.

## Figures and Tables

**TABLE 1:** Demographics, Comorbidities, Symptoms, and Outcomes Among Diagnosed and Hospitalized COVID-19 Children and Adolescents (<18 y of Age)

	At Least 1 y of Previous Observation Available								No Previous Observation Time Available						
	Diagnosed				Hospitalized				Diagnosed					Hospitalized	
	SIDIAP (Spain)	IQVIA LPD (France)	CU-AMC HDC (United States)	IQVIA Open Claims (United States)	Optum EHR (United States)	HIRA (South Korea)	IQVIA Open Claims (United States)	Optum EHR (United States)	CUIMC (United States)	Premier (United States)	IQVIA DA (Germany)	HEALTHVERITY (United States)	STARR-OMOP (United States)	CUIMC (United States)	Premier (United States)
	*n* = 4494	*n* = 695	*n* = 189	*n* = 176 668	*n* = 10 166	*n* = 251	*n* = 6499	*n* = 752	*n* = 425	*n* = 21 148	*n* = 1150	*n* = 27 038	*n* = 185	*n* = 210	*n* = 2057
Age, y															
0–4	31.2	11.4	11.6	15.3	16.0	17.1	23.2	27.3	60.2	30.5	29.4	16.6	38.4	57.1	54.2
5–9	28.7	23.2	23.8	23.5	21.0	16.3	24.8	19.4	15.5	18.5	28.2	20.5	16.8	15.2	12.1
10–14	24.0	36.3	23.3	31.0	28.5	33.5	25.5	25.4	13.2	24.0	23.0	32.4	19.5	15.2	13.0
15–19	16.1	29.2	41.3	30.3	34.5	33.1	26.4	27.9	11.1	26.9	19.5	30.4	25.4	12.4	20.7
Sex															
Female	47.2	53.2	48.1	50.2	50.1	49.4	48.8	50.0	44.7	50.3	47.7	49.2	50.8	44.8	48.5
Male	52.8	46.6	51.9	49.8	49.9	50.6	51.2	50.0	55.3	49.7	52.3	50.8	49.2	55.2	51.5
SARS-CoV-2–positive test result	2.1	—	—	—	70.4	—	—	48.1	37.2	—	—	78.3	42.7	55.2	—
Comorbidities^a^															
Autistic disorder	0.9	—	—	1.0	1.2	—	2.9	2.5	—	—	—	—	—	—	—
Neonatal disorder	2.3	—	—	0.4	0.5	—	2.4	2.4	—	—	—	—	—	—	—
Neurodevelopmental disorder	7.4	1.0	7.4	7.1	8.2	2.4	15.4	16.5	—	—	—	—	—	—	—
Asthma	10.1	18.7	16.4	28.1	20.6	35.1	34.1	29.3	—	—	—	—	—	—	—
Obesity	9.5	1.9	—	9.5	19.0	—	18.0	19.9	—	—	—	—	—	—	—
Heart disease	1.9	1.2	—	6.9	6.7	3.6	29.7	15.4	—	—	—	—	—	—	—
Malignant neoplasm excluding nonmelanoma skin cancer	0.3	—	—	1.2	3.4	—	9.2	10.8	—	—	—	—	—	—	—
Hypertension	0.3	—	—	3.2	1.5	—	21.0	4.9	—	—	—	—	—	—	—
Type 1 diabetes mellitus	0.2	—	—	0.4	0.3	—	2.4	1.1	—	—	—	—	—	—	—
Attention-deficit/hyperactivity disorder	2.2	—	—	4.9	5.7	—	5.8	9.7	—	—	—	—	—	—	—
Chromosomal disorder	0.4	—	—	0.4	0.5	—	3.4	2.1	—	—	—	—	—	—	—
Congenital malformation	10.8	—	—	3.2	4.0	4.4	14.0	11.4	—	—	—	—	—	—	—
Congenital heart disease	0.2	—	—	0.4	0.5	—	3.0	2.4	—	—	—	—	—	—	—
Prematurity of infant	1.0	—	—	0.3	0.6	—	2.0	1.6	—	—	—	—	—	—	—
Symptoms at the index date															
Fever	4.8	10.1	7.9	8.4	7.8	10.0	10.7	14.1	26.4	21.8	4.2	6.2	17.8	28.1	17.3
Cough	4.7	8.2	8.5	7.0	6.8	5.6	4.3	6.6	8.2	13.0	2.7	6.3	9.7	2.9	2.2
Dyspnea	0.3	—	—	1.7	1.1	9.6	7.8	5.1	4.7	4.5	—	0.7	8.6	10.0	8.1
Malaise or fatigue	—	1.9	—	1.1	0.9	—	1.2	1.1	—	1.2	—	1.0	—	—	1.1
Myalgia	—	—	—	0.5	0.6	—	0.4	1.1	—	1.4	—	0.2	—	—	0.3
Anosmia, or hyposmia, dysgeusia	—	—	—	0.8	1.1	—	—	—	—	1.5	—	0.5	—	—	—
Gastrointestinal tract symptoms	12.5	4.3	—	2.6	2.9	8.0	13.2	10.2	8.2	6.5	1.3	0.5	10.3	9.0	13.2
Diarrhea	4.0	—	—	1.1	1.3	—	3.6	3.5	1.9	2.7	—	0.3	—	—	4.1
Vomiting	1.8	1.9	—	1.4	1.4	2.4	8.7	4.8	4.5	3.4	0.8	0.2	7.0	5.7	4.9
Nausea	1.4	1.9	—	1.1	1.1	2.0	4.5	3.6	—	2.2	0.8	0.1	—	—	1.6
Bronchiolitis	0.5	—	—	0.3	0.3	—	3.9	3.7	8.9	2.1	—	0.1	9.7	11.4	14.4
30-d outcomes after hospitalization															
Sepsis	—	—	—	—	—	—	9.4	3.9	—	—	—	—	—	3.3	10.1
ARDS	—	—	—	—	—	—	13.2	6.2	—	—	—	—	—	8.1	16.5
Cardiac arrhythmia	—	—	—	—	—	—	8.2	3.1	—	—	—	—	—	9.0	5.9
Bleeding	—	—	—	—	—	—	3.3	1.2	—	—	—	—	—	—	4.1
30-d outcomes															
Death	—	—	—	—	—	—	—	—	—	0.0	—	—	—	—	—
Hospitalization episodes	1.4	—	—	3.5	7.6	—	—	—	33.2	10.2	—	0.3	30.8	—	—
Pneumonia	3.1	—	—	1.8	1.0	6.8	15.6	7.6	4.5	0.0	—	0.1	—	7.6	—
MIS-C	—	—	—	0.2	0.2	—	3.5	2.1	3.1	0.3	—	0.0	—	7.6	2.2
Hypoxemia	—	—	—	1.1	0.8	—	23.6	9.3	6.8	2.6	—	0.1	13.5	12.9	22.3

Proportions presented among patients diagnosed with COVID-19 or hospitalized with COVID-19 by database (column percentage). Hospitalized children and adolescents could also be included in the diagnosed cohorts. Children aged <1 y were excluded when at least 1 y of previous observation time was required. —, data not available or below the minimum cell count required (5 individuals).

a Comorbidities are reported only in those databases with at least 1 y of previous observation time.

## WHAT’S KNOWN ON THIS SUBJECT:

Most coronavirus disease 2019 (COVID-19) studies were targeted at adults, and data concerning children and adolescents are limited. Clinical manifestations of COVID-19 are generally milder in the pediatric population compared with adults, and hospitalization for COVID-19 affects mostly children with preexisting comorbidities.

## WHAT THIS STUDY ADDS:

Complications including hospitalization, hypoxemia, and pneumonia were more frequent in children and adolescents with COVID-19 than with influenza. Dyspnea, anosmia, and gastrointestinal symptoms could help differential diagnosis. A wide range of medications was used for the inpatient management of pediatric COVID-19.

## ABBREVIATIONS

ARDS: acute respiratory disease syndrome
CHARYBDIS: Characterizing Health Associated Risks, and Your Baseline Disease In Severe acute respiratory syndrome coronavirus 2
COVID-19: coronavirus disease 2019
CU-AMC HDC: Colorado University Anschutz Medical Campus Health Data Compass
CUIMC: Columbia University Irving Medical Center
DA: Data Analyzer
EHR: electronic health record
HIRA: Health Insurance Review & Assessment Service
LPD: Longitudinal Patient Data
MIS-C: multisystem inflammatory syndrome in children
SARS-CoV-2: severe acute respiratory syndrome coronavirus 2
SIDIAP: Information System for Research in Primary Care
STARR-OMOP: STAnford Research Repository Observational Medical Outcomes Partnership
